# HAfTs are novel lncRNA transcripts from aflatoxin exposure

**DOI:** 10.1371/journal.pone.0190992

**Published:** 2018-01-19

**Authors:** B. Alex Merrick, Justin S. Chang, Dhiral P. Phadke, Meredith A. Bostrom, Ruchir R. Shah, Xinguo Wang, Oksana Gordon, Garron M. Wright

**Affiliations:** 1 Biomolecular Screening Branch, Division National Toxicology Program, National Institute of Environmental Health Sciences, Research Triangle Park, North Carolina, United States of America; 2 Sciome, LLC, Research Triangle Park, North Carolina, United States of America; 3 Genomics Laboratory, David H. Murdock Research Institute, Kannapolis, North Carolina, United State of America; Florida Atlantic University, UNITED STATES

## Abstract

The transcriptome can reveal insights into precancer biology. We recently conducted RNA-Seq analysis on liver RNA from male rats exposed to the carcinogen, aflatoxin B1 (AFB1), for 90 days prior to liver tumor onset. Among >1,000 differentially expressed transcripts, several novel, unannotated Cufflinks-assembled transcripts, or HAfTs (**H**epatic **Af**latoxin **T**ranscripts) were found. We hypothesized PCR-cloning and RACE (rapid amplification of cDNA ends) could further HAfT identification. Sanger data was obtained for 6 transcripts by PCR and 16 transcripts by 5’- and 3’-RACE. BLAST alignments showed, with two exceptions, HAfT transcripts were lncRNAs, >200nt without apparent long open reading frames. Six rat HAfT transcripts were classified as ‘novel’ without RefSeq annotation. Sequence alignment and genomic synteny showed each rat lncRNA had a homologous locus in the mouse genome and over half had homologous loci in the human genome, including at least two loci (and possibly three others) that were previously unannotated. While HAfT functions are not yet clear, coregulatory roles may be possible from their adjacent orientation to known coding genes with altered expression that include 8 HAfT-gene pairs. For example, a unique rat HAfT, homologous to Pvt1, was adjacent to known genes controlling cell proliferation. Additionally, PCR and RACE Sanger sequencing showed many alternative splice variants and refinements of exon sequences compared to Cufflinks assembled transcripts and gene prediction algorithms. Presence of multiple splice variants and short tandem repeats found in some HAfTs may be consequential for secondary structure, transcriptional regulation, and function. In summary, we report novel, differentially expressed lncRNAs after exposure to the genotoxicant, AFB1, prior to neoplastic lesions. Complete cloning and sequencing of such transcripts could pave the way for a new set of sensitive and early prediction markers for chemical hepatocarcinogens.

## Introduction

Aflatoxin B1 (AFB1) is a naturally occurring mycotoxin produced by *Aspergillus flavus* and *Aspergillus parasiticus* and is a contaminant of grains, animal and pet feed and a variety of consumer food products [1, 2]. It is particularly prevalent in developing countries where grain storage occurs in hot and unsheltered conditions [3]. The reason for concern is that AFB1 is metabolically activated to an epoxide to form DNA-adducts (e.g. genotoxic) and is a potent liver carcinogen in many species [4]. Rats are a sensitive species since continuous, chronic ingestion of AFB1 can cause up to 100% incidence of multiple hepatocellular carcinomas [5–8]. Differences in metabolic activation and detoxication enzymes contributed to an early understanding of susceptibility to AFB1 hepatocellular carcinomas among various species and gender differences (males are more sensitive than females) [9]. Continued work on AFB1 carcinogenesis has led to discovery of compounds that protect against AFB1 liver tumors such as oleanane triterpenoids as activators of Keap-Nrf2 and other pathways that hold great promise for future chemoprevention strategies [10].

The DNA adduct of the AFB1 epoxide, AFB1-N^7^-Gua, primarily forms G:C to T:A mutations (primarily transversions) in rodents and humans [11, 12]. Though molecular adducts of AFB1 are well understood, common driver mutations, in addition to Tp53 [12, 13], that comprehensively explain liver tumor formation remain elusive due to the complexity and heterogeneity of hepatocellular carcinomas [14]. Genome-wide expression analyses have been useful in identifying commonly altered biological pathways and processes during AFB1 carcinogenesis in animal and cultured cell models. AFB1-induced changes in liver have been observed in PXR and Cyp isoforms and detoxication pathways [15–19]; anti-oxidant and Nrf2-related pathways [19, 20]; cell cycle, proliferation and p53-dependent pathways [21–23]; as well as immune, cell adhesion and signal transduction processes [18, 19, 23]. RNA-Seq analysis has widened the number of cellular pathways and processes affected by AFB1 exposure in rodent, poultry and porcine models [24–31] including miRNAs [32] and lncRNAs [33]. The association of lncRNA changes to AFB1 exposure and development of hepatocellular carcinomas [33] is a new finding. In this study [33], a 62-week exposure to AFB1 caused most rats to develop hepatocellular carcinomas while one-quarter of those similarly exposed did not, and were called–‘aflatoxin-resistant’ rats. When compared to controls, RNA-Seq revealed a large group of lncRNAs that were highly expressed only in AFB1 hepatocellular carcinomas, and was coincident with up-regulated cancer-related transcripts related to cell cycle, apoptosis, and DNA repair. Such cancer-related transcripts were down-regulated in resistant rats when specific lncRNA clusters were highly expressed. Expression of lncRNAs was correlated with altered protein-coding transcripts controlling phosphorylation, stress response, T cell and leukocyte activation as well as cell cycle, apoptosis, and DNA damage response. Results suggested a coordinated expression of protein-coding and lncRNA genes in hepatocellular carcinogenesis and AFB1 resistance.

In our initial AFB1 study, RNA-Seq analysis was performed on liver RNA extracted from male rats subchronically exposed to 1 ppm of AFB1 in feed. Robust changes were observed in the preneoplastic hepatic transcriptome where AFB1 altered the expression of over 1000 genes [26]. Pathway analysis among differentially expressed genes (DEGs) showed activation of Ahr, Nrf2, GSH, xenobiotic, cell cycle (e.g. E2f1), extracellular matrix, and cell differentiation networks, consistent with pathways leading to AFB1-induced carcinogenesis. In addition, we reported differential expression of several completely unannotated novel transcripts (twenty-eight) not in the RefSeq or Ensembl databases that were assembled by the Cufflinks algorithm from RNA-Seq reads. We termed these unannotated novel transcripts as ‘HAfTs’–Hepatic Aflatoxin Transcripts–and hypothesized they were relevant to the malignant transformation process from AFB1 exposure. The objective of the current study was to validate, refine and try to identify these novel transcripts from AFB1 RNA by Sanger sequencing of cDNA after PCR cloning and by 5’- and 3’-RACE (rapid amplification of cDNA ends). While a few of these transcripts are now identified as known genes, sequencing indicates that most HAfTs are lncRNAs including several that remain unannotated and novel.

## Materials and methods

### Animal care and tissue collection

Animal sourcing, care, chemical exposure and tissue collection have been previously described in prior published work [34] and details are briefly provided here. Male F344/N rats at 8 to 10 weeks of age were procured from Taconic Farms (Germantown, NY). Animals were maintained in an AAALAC-accredited facility and quarantined for 10 to 14 days for acclimation prior to study. Animals were randomly assigned to control (NTP 2000 diet) and treatment groups for exposure to 1ppm aflatoxin B1 (CAS No. 1162-65-8) incorporated into feed. Rats were maintained on a 12:12 light and dark cycle starting at 6AM to 6PM for light. Rats were housed at three per cage and allowed free access to food and water. During exposure in the study, animals were monitored weekly for body weight, and twice daily for clinical and physical health observations, and food and water consumption. On the day of sacrifice, animals were necropsied between 8 and 10AM and anesthetized with isoflurane. Rats were euthanized under anesthesia by exsanguination. Left and median liver lobes were removed, minced, quickly flash frozen and then stored at -80°C.

All experiments were performed in accordance with the Animal Welfare Act and the U.S. Public Health Service Policy on Humane Care and Use of Laboratory Animals after review and approval by the Institutional Animal Care and Use Committee (IACUC) of the National Institute of Environmental Health Sciences at Research Triangle Park, NC.

### Novel transcripts

Findings of novel liver transcripts by RNA-Seq analysis were described previously [26] and have been validated here by PCR or RACE (Rapid Amplifications of cDNA Ends). Briefly, RNA was extracted from livers of F344 male rats (Qiagen RNeasy Midi Kit, Germantown, MD) after 90-day feed exposure to 1ppm AFB1 or control feed at 4 animals per group. RNA fragmentation and paired end RNA-Seq analysis was performed after polyA enrichment. An Illumina GX-II instrument sequencing produced about 30–37 million paired end 100bp reads per animal. RNA-Seq reads were aligned using TopHat and assembled into transcripts by Cufflinks and DESeq was used to test for differential expression between AFB1 and control groups. Novel DEG transcripts were found that did not have RefSeq or Ensembl annotation (coordinates in S1 Table). RNA-Seq procedures are described in greater detail in prior work [26].

Confirmation and further refinement of HAfT transcripts was carried out using primers as outlined in S2 Table that were based on sequences from Cufflinks assembled transcripts. PCR or 5’-RACE/3’-RACE reactions were used to screen for single amplicons corresponding to HAfT sequences. Amplification products were separated by agarose gels, detected by ethidium bromide (EtBr) staining, and cDNA was extracted from gel slices. Occasionally, PCR produced two amplicons (such as with HAfT6) where each was sequenced as a possible splice variant. TA cloning of PCR amplicons and Phi29 DNA polymerase-based rolling circle amplification were used to screen colonies by Sanger sequencing. 5’- or 3’-RACE amplicons were captured by directional cloning prior to Sanger sequencing. Overlapping Sanger sequences were assembled into consensus sequences using BLAT alignment to Cufflinks transcripts in the UCSC genome browser for each transcript and associated variants. These procedures are described in more detail in the sections below.

#### PCR amplification, cloning, and sequencing

The objectives of PCR-cloning and RACE sequencing were to validate HAfTs and to improve transcript structure (e.g. multiple exons occurring within a Cufflinks exon). Primers were generally designed from exon sequences and did not span exon-exon boundaries since exonic features were sometimes unclear in Cufflinks transcripts. When possible, primers were designed for transcript walking to determine the location of exons. Amplicons were produced by PCR with sequence-specific primers (S2 Table) using reverse transcriptase (RT) oligo-dT reactions prepared according to the manufacturer’s protocol (Invitrogen, Carlsbad, CA). Sequence-specific primers were derived from Cufflinks assembled sequences. PCR amplification reaction mixtures were in a 15 μL volume comprised of 7.5 μL of 2X Hot Start Sweet Master Mix (SABiosciences, Frederick, MD, USA), 0.6 μL of primers (100 nM final concentration), 1 μL of RT reaction (1 μL cDNA/20 μL RT reaction), and RNase-free water. Thermocycler conditions for PCR were 95°C for 10 min for polymerase activation, 95°C for 30 s, 55 to 60°C for 30 s, and 72°C for 30 s for 35 cycles, followed by a 7 min extension at 72°C. PCR products were separated by 2% agarose gel electrophoresis and detected by ethidium bromide fluorescence. Briefly, amplicons were gel purified in 2% agarose, cut out, melted, and purified on silica gel spin columns (Qiagen, Valencia, CA, USA) and TOPO TA cloned into chemically competent *E*. *coli* (TopTen cells, Invitrogen) according to the manufacturer’s protocol. Transformed cells were selected for positive clones on 50 μg/mL Kanamycin LB agar dishes containing X-gal. Template DNA from positive clones was amplified using a Phi29 DNA polymerase system (illustra TempliPhi Kit; GE Healthcare Life Sciences, Pittsburgh, PA) prior to Sanger sequencing of plasmids using forward and reverse M13 sequencing primers.

#### RACE amplification

Primers were designed for RACE amplification (Clontech-Takara, Mountain View, CA) using the SMARTer^®^ RACE 5’/3’ kit per manufacturer’s instructions. Gene-specific RACE primers were designed from Cufflinks assembled sequences. Note that the 5’-end of gene-specific RACE primers contained the sequence, gattacgccaagctt to facilitate In-Fusion^®^ directional cloning (Clontech). Four sets of primers were made for each HAfT transcript: two primer sets were targeted to the 5’-end and two primer sets targeted the 3’-end of each transcript (S2 Table). The protocol is briefly summarized. A 1μg total RNA amount was used for each cDNA synthesis reaction. RACE cDNA reactions contained 1μg RNA, either 1μl of 5’-CDS Primer A or 1μl of 3’CDS Primer A and RNase free water to 11μl volume, followed by mixing and denaturation at 72°C for 3 min and cooling for 2 min. The 5’-RACE reaction then received 1μl of SMARTer IIA oligonucleotide and the 3’-RACE reaction received 1μl of RNase-free water so each reaction was at a 12μl volume. Each RACE reaction received 8μl of Master Mix [4μl 5X Buffer, 0.5μl 100mM DTT, 1μl dNTPs (20mM), 0.5ul RNase Inhibitor (40U/μl) and 2ul SMARTScribe^™^ reverse transcriptase (100U)] for a total volume of 20μl. cDNA synthesis proceeded at 42°C for 90 min, followed by reaction termination at 70°C for 10min. Each 50μl RACE reaction, contained 25μl of 2X SeqAmp buffer, 15.5μl RNase free water, 2.5μl of 5’- or 3’-RACE cDNA, 5μl of 10X Universal Primer Mix, 1μl of 5’ or 3’ Gene Specific Primer (10μM) and 1μl of SeqAmp DNA polymerase. RACE cycling conditions were for 25 cycles at 94°C for 30 sec, 68°C for 30 sec, and 72°C for 3 min. In-Fusion^®^ directional cloning of RACE products was performed using Stellar^™^ competent *E*. *coli* (Clontech) prior to Sanger sequencing.

### BLAST alignment and homology analysis

Alignment searches were performed with NCBI’s BLAST suite. HAfT sequences were designated as ‘experimental’ (Sanger sequencing), ‘computational’ (Cufflinks) or ‘Known Loc” (known sequence at a genomic locus in RefSeq) as described by column headings in Table 1. Sequences were aligned to rat, mouse, and human RNA or DNA by chromosome using command line blastn version 2.2.29. For example, the blastn command line parameters used while aligning to mouse RNA were: ‘-evalue 0.01 -remote -entrez query "Mus musculus[organism] AND biomol_rna[Properties]‴ -db nt. The blastn parameters used while aligning to mouse chromosomes were: ‘-evalue 0.01 -remote -entrez_query "Mus musculus[organism]‴ -db chromosome".

**Table 1:** Summary of NCBI search alignments of HAfT consensus sequences.

HAfT#^a^	RefSeq	Experimental Sequence	Cufflinks Assembly	PCR	RACE
HAfT1	LOC103691380	HAfT1_809nt	Cufflinks_00006229_2440nt	**•**	**•**
HAfT2	LOC102549099	HAfT2_209nt	Cufflinks_00021611_1424nt	**•**	
HAfT3	LOC103690347	HAfT3_637nt	Cufflinks_00005778_832nt		**•**
HAfT4	LOC102549932_VariantX1	HAfT4_598nt	Cufflinks_00014924_1326nt		**•**
HAfT5	LOC103693988	HAfT5_410nt	Cufflinks_00024199_2117nt		**•**
HAfT6	LOC102552829_VariantX1	HAfT6_VariantX1_1720nt	Cufflinks_00024116_528nt	**•**	**•**
	LOC102552829_VariantX2	HAfT6_VariantX2_1640nt	Cufflinks_00024274_5559nt		
HAfT7	LOC102555869_VariantX1;	HAfT7_VariantX1_383nt	Cufflinks_00037591_4007nt		**•**
	LOC102555869_VariantX2	HAfT7_VariantX2_656nt			
		HAfT7_VariantX3_749nt			
HAfT8	LOC102554927	HAfT8_VariantX1_1587nt	Cufflinks_00026037_1707nt	**•**	**•**
	LOC103694063	HAfT8_VariantX2_1501nt	Cufflinks_00025137_8415nt		
		HAfT8_VariantX3_663nt			
HAfT9	LOC103694221	HAfT9_495nt	Cufflinks_00027620_1621nt		**•**
HAfT10	LOC102554057_VariantX6	HAfT10_VariantX1_375nt	Cufflinks_00032581_482nt		**•**
		HAfT10_VariantX2_342nt	Cufflinks_00030744_1853nt		
HAfT11	LOC102547196	none	Cufflinks_00029308_1820nt		
HAfT12	LOC102555623_VariantX2	none	Cufflinks_00032864_1175nt		
			Cufflinks_00033179_2265nt		
HAfT13	LOC102548205	none	Cufflinks_00035253_1571nt		
HAfT14	LOC100910558_Variant1	none	Cufflinks_00047409_1350nt		
	LOC100910558_Variant2				
	LOC100910558_Variant3				
HAfT15	LOC102552388_VariantX1	none	Cufflinks_00048965_3008nt		
	LOC102552388_VariantX2				
HAfT16	LOC102553833	none	Cufflinks_00055698_7580nt		
			Cufflinks_00057006_3621nt		
HAfT17	LOC108349448_VariantX1	none	Cufflinks_00006612_258nt		
	LOC108349448_VariantX2				
HAfT18	NOVEL lncRNA	none	Cufflinks_00036996_5428nt		
HAfT19	NOVEL lncRNA	HAfT19_VariantX1_477nt	Cufflinks_00026127_939nt		**•**
		HAfT19_VariantX2_447nt			
		HAfT19_VariantX3_308nt			
HAfT20	NOVEL lncRNA	HAfT20_508nt	Cufflinks_00027672_737nt		**•**
HAfT21	NOVEL lncRNA	HAfT21_VariantX1_139nt	Cufflinks_00034634_2443nt		**•**
		HAfT21_VariantX2_223nt			
HAfT22	NOVEL lncRNA	HAfT22_VariantX1_760nt	Cufflinks_00053773_2795nt		**•**
		HAfT22_VariantX2_563nt	Cufflinks_00078728_4974nt		
HAfT23	LOC108351462	HAfT23_VariantX1_873nt	Cufflinks_00053917_915nt	**•**	**•**
		HAfT23_VariantX2_1053nt	Cufflinks_00053916_3238nt		
			Cufflinks_00078399_8748nt		
HAfT24	NOVEL lncRNA	HAfT24_499nt	Cufflinks_00007062_1742nt		**•**
HAfT25	Pvt1_VariantX2	HAfT25_1501nt	Cufflinks_00054567_2279nt	**•**	**•**
			Cufflinks_00054102_629nt		
HAfT26	Cyp2c24_NM001271354	none	Cufflinks_00006202_425nt		
HAfT27	Rps27l_NM001276477	none	Cufflinks_00056890_307nt		
			Cufflinks_00055299_577nt		

^a^A summary is presented for database searches of HAfT consensus experimental sequences and Cufflinks assembly sequences from rat transcripts. Experimental sequences were determined by Sanger sequencing of PCR and/or RACE products as indicated (•). HAfT11 to HAfT18 did not have Sanger data, so alignment searches were conducted on Cufflinks assembled transcripts. Searches suggested HAfT11 to HAfT17 were lncRNAs while HAfT18 was not annotated. Similarly, experimental sequences for HAfT19, 20, 21, 22, and 24 were unannotated and classified as ‘NOVEL lncRNA’. HAfT25 most closely aligns with a predicted model ncRNA for rat Pvt1 (variant X2), while there are 21 possible predicted variants for rat Pvt1 (S2 Fig). Alignment searches of HAfT26 and 27 revealed these transcripts are, respectively Cyp2c24 and Rps27l, as rat Ref-Seq protein-coding genes. For some HAfTs (e.g. HAfT10), multiple Cufflinks transcript variants were needed to account for experimental Sanger data or lncRNA sequence alignments. Similarly, some HAfTs (e.g. HAfT14) aligned with multiple predicted lncRNA variants (e.g. LOC100910558_Variant1, 2, 3).

For alignment to RNA sequences, the top search result with an evalue <0.01 was reported. RNA transcripts can have multiple exons aligning to different non-contiguous regions of a chromosome. So, for alignment to a mouse or human chromosome, the top 10 alignments with an evalue<0.01 retained the non-redundant and non-overlapping alignments to the same chromosome and were then summarized as the non-contiguous alignment of the RNA transcript on a specific chromosome. The NCBI RNA identity or chromosomal location for each sequence from the BLAST result was recorded. The percentage alignment by coverage and identity from the BLAST result were described. A 30% threshold was set for transcript homology in rat, mouse, and human species.

### Cloverleaf structure plots

ncRNA functions are often related to their secondary structure which can be computationally modeled using predicted minimum free energy (MFE) structures. Secondary shapes of RNA were visualized by ‘cloverleaf’ structures using the RNAfold Server from the ViennaRNA services at http://rna.tbi.univie.ac.at/. The RNAfold server algorithms predict the minimum free energy associated with a most likely structure for a submitted sequence and provides a graphical output based on base pair probabilities [35]. HAfT19 transcripts were analyzed using the MFE and partition function algorithms that avoided isolated base pairs and included default output options. For comparison among HAfT variants, differences in transcript length were normalized by calculating an adjusted MFE (AMFE), as MFE divided by the sequence length X 100 [36].

### Genomic synteny and orientation

The sequences of rat HAfTs were aligned in mouse (GRCm38/Annotation 106) and human genomes (GRCh38/Annotation 108). The order of surrounding genes flanking each HAfT locus (using longest sequence) from *Rattus norvegicus* Rnor_6.0/Annotation 106 was compared for conserved synteny in the mouse or human genomes. In addition, HAfT transcripts were examined at their genomic locus for location on forward (+) or reverse (-) DNA strands to determine their relative orientation to immediately adjacent (closest to either 5’- or 3’-end), annotated genes. Changes in adjacent gene expression were noted as a potential HAfT-gene expression pair for coregulation.

### Conserved motifs and tandem repeat sequences

HAfT sequences were searched in the Conserved Domains Database (CDD) https://www.ncbi.nlm.nih.gov/Structure/cdd/wrpsb.cgi for polypeptide structural and functional motifs since open reading frames in lncRNA are sometimes translated [37].

RepeatMasker at http://www.repeatmasker.org/cgi-bin/WEBRepeatMasker was used to screen for interspersed repeats and low complexity DNA sequences [38, 39].

## Results

### HAfT transcripts are lncRNAs

**Fig 1:**
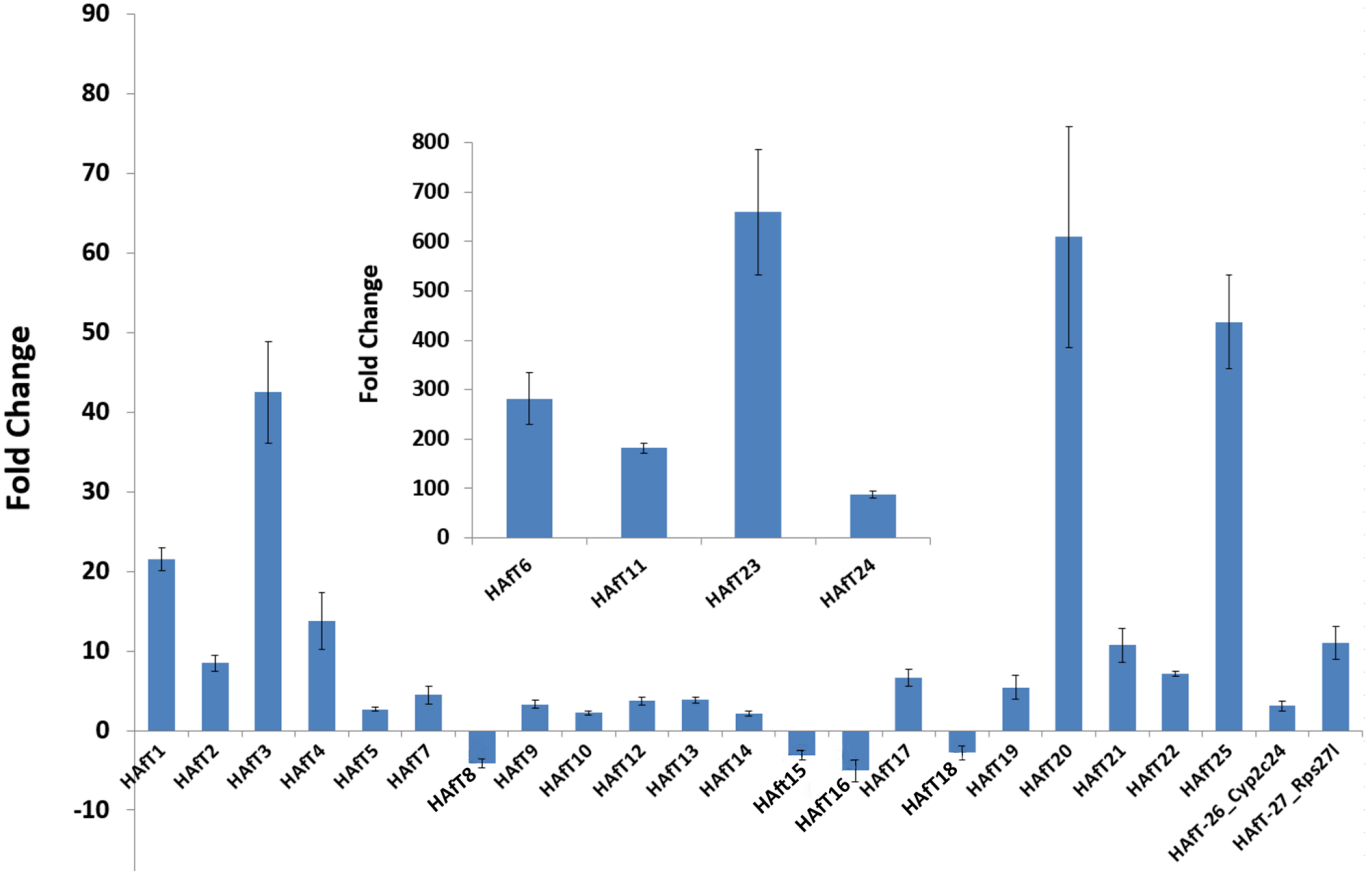
HAfT fold changes from AFB1 exposure. RNA-Seq was performed on liver RNA from male rats exposed for 90 days to 1 ppm AFB1. HAfTs were originally assembled as unannotated transcripts from this RNA-Seq data as previously described [26]. Shown here, HAfTs met a two-fold change threshold at p<0.05 (mean ± rSEM). NCBI databases now list HAfT26 and HAfT27 as protein-coding genes, Rps27l and Cyp2c24. HAfT-6, -11, -23 and -24 were placed on a separate scale (see inset) due to the large magnitude of fold change.

Since publication of the initial set of 28 unannotated transcripts [26], HAfT sequences were re-queried in NCBI and Ensembl databases before designing primers. Alignment searches revealed that there were twenty-five HAfT transcripts that remained for further investigation after accounting for recent annotation of Cyp2c24 and two similar transcripts of Rps27l. Fig 1 shows the fold changes of all HAfT transcripts.

**Fig 2:**
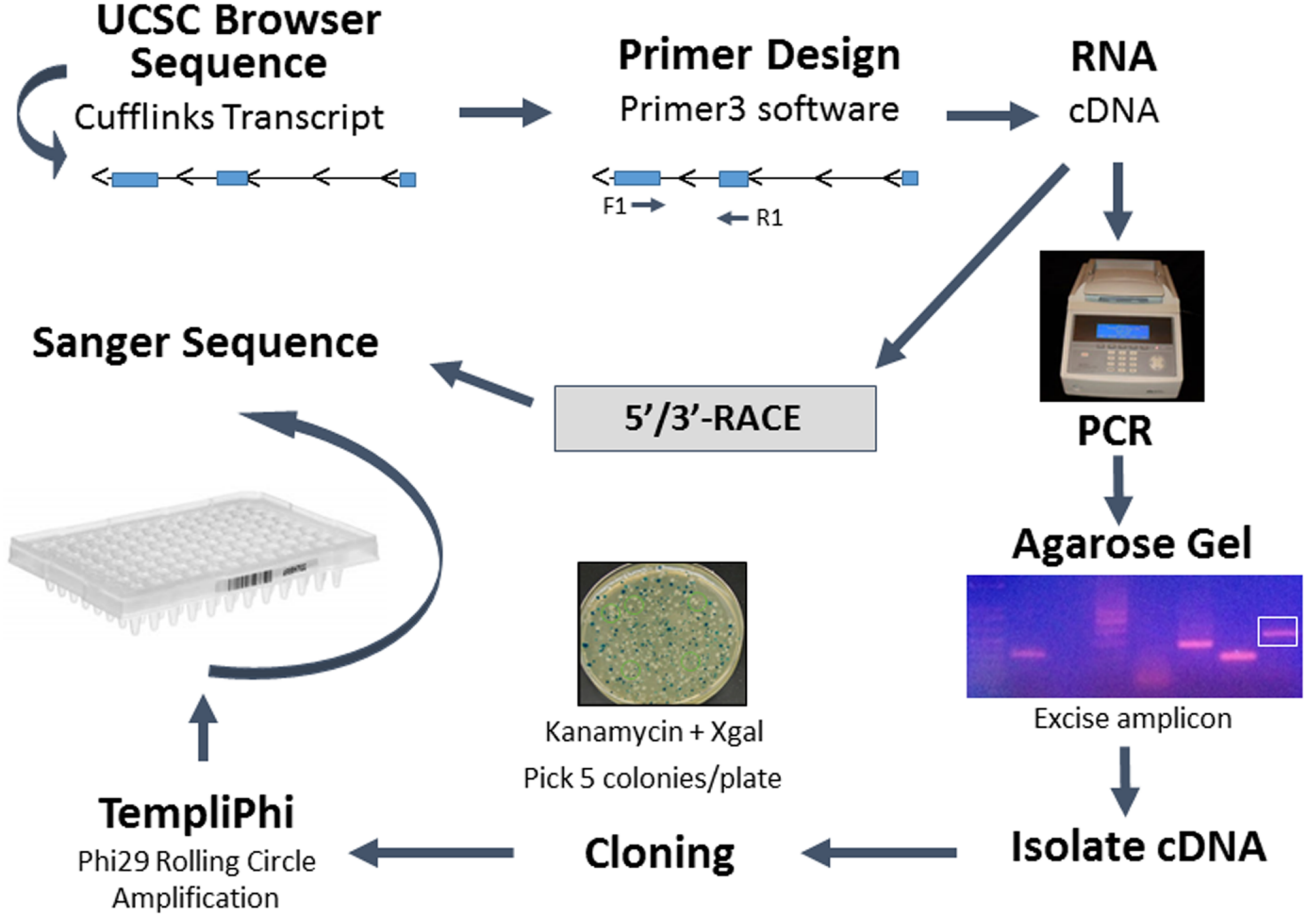
Workflow for PCR and RACE, cloning and sequencing. See Methods for further details.

A workflow (Fig 2) was designed to investigate HAfT1 to HAfT25 by developing primers from sequences of Cufflinks assembled transcripts. Initially, ten HAfT transcripts were selected for PCR-cloning based on high expression or more possible exons than Cufflinks predictions. RNA from two independent animals were reverse transcribed to cDNA and PCR was performed. Initial screening of PCR primer sets (S2 Table) showed several HAfT transcripts (HAfT3, 6, 8, 23 and 25) could be Sanger sequenced and assembled. Further, additional primers were designed for 5’- and 3’-RACE (S2 Table) that produced amplicons for Sanger sequencing and were assembled into consensus sequences. HAfTs from PCR and RACE data were assigned NCBI accession numbers (S3 Table), noting transcript variants when they occurred. HAfT chromosomal locations are described in S1 Table.

Consensus sequences of HAfT1 to HAfT25 were searched and aligned to annotated transcripts in the NCBI database. Table 1 shows many HAfT sequences aligned with recently predicted rat lncRNAs. In the present study, Sanger sequencing data were produced for 16 HAfT transcripts by PCR (HAfT3, 6, 8, 23 and 25) and RACE (HAfT1, HAfT3-10 and HAfT19-25).

HAfT1 to 10 have experimental sequencing data that align with predicted lncRNAs (e.g. LOC102549099) as indicated in the RefSeq column of Table 1, that we have experimentally validated for the first time. While Sanger data were not produced for HAfT11 to 17, the Cufflinks assembled sequences could be sufficiently aligned to identify them as predicted NCBI rat lncRNAs. However, HAfT18, 19, 20, 21, 22 and 24 continue to be unannotated, and are proposed as novel lncRNAs. For some HAfTs (e.g. HAfT10), more than one Cufflinks transcript was needed to account for the predicted lncRNA sequence.

### Novel HAfT transcripts

**Fig 3:**
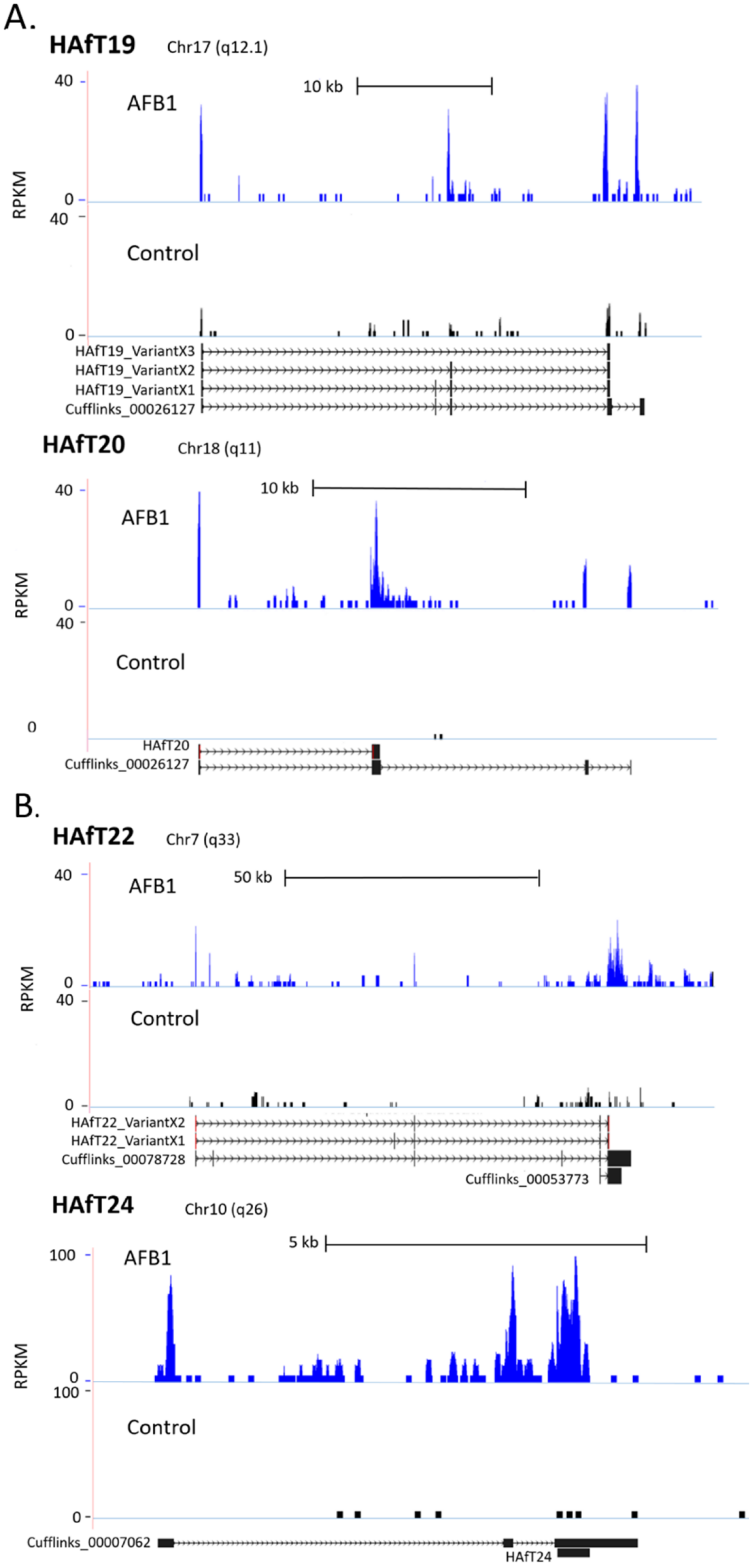
Novel HAfTs. Four novel, unannotated HAfTs and variants are shown in the UCSC genome browser. AFB1 treatment resulted in an increased number of RNA-Seq reads at specific genomic loci that enabled assembly of Cufflinks transcripts. Primers were designed from Cufflinks transcripts and PCR produced amplicons for portions of HAfT19 and 20 in Panel A, and HAfT22 and 24 in Panel B. Additional variants may exist for these novel HAfT transcripts.

Fig 3 shows a subset of four novel, unannotated HAfTs and variants in UCSC genome browser format. AFB1 elicited increased reads at these HAfT specific loci enabling transcript detection after Cufflinks assembly. From these assembly sequences, we designed primers for PCR and sequenced the products to confirm HAfT19, HAfT20, HAfT22 and HAfT24. Fig 3 Panel A shows three experimental variants were found for HAfT19 from which two variant transcripts, Variant_X2 and X3, may result from exon skipping. Two exons in HAfT20 were confirmed by Sanger data although additional variants may exist. HAfT22 in Fig 3 Panel B also shows how Sanger data can refine transcript structure and reveal variants. HAfT22 Variant_X1 shows an exon not predicted by Cufflink assemblies.

**Fig 4:**
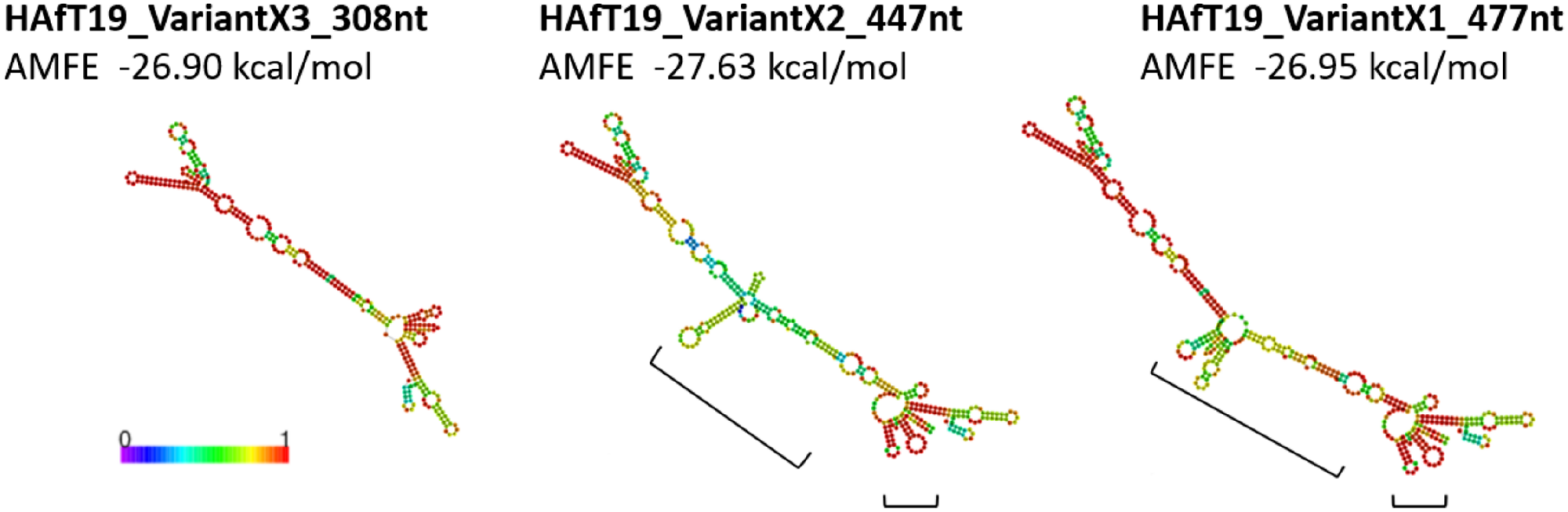
MFE clover plots. Minimum free energy (MFE) cloverleaf models were generated for HAfT19 variants. Each circle represents a base, and color-coding indicates the base-pairing probability, with 0 (blue) to 1 (red) representing low to high pairing probabilities. The adjusted MFE (AMFE) normalizes for differing transcript length. Brackets show predicted differences in secondary structure when comparing the shortest HAfT19_VariantX3 after addition of one exon (HAfT19_VariantX2) or two exons (HAfT19_VariantX1). See text for further details.

The occurrence of splice variants can have observable effects upon ncRNA secondary structure, that may produce changes in variant function. We chose closely related variants of HAfT transcripts to illustrate this point (Fig 4). The three variants of HAfT19 consist of 4 exons for VariantX1_477nt with 3 exons for VariantX2_447nt and 2 exons for VariantX3_308nt. Minimum free energy (MFE) cloverleaf models were generated for HAfT19 variants. MFE values of HAfT19 variants X1, X2 and X3 (-83.00 kcal/mol, -123.5 kcal/mol, -128.3 kcal/mol, respectively) were adjusted to AMFE values to account for differing transcript lengths and normalized per 100 nt. AMFEs for Variants X1, X2 and X3 were generally comparable (Fig 4). The simplest structure for the two exon VariantX3 transcript (Fig 4) can be modified by the additional exons contained in transcript VariantX2 and VariantX1 sequences to create new stem-loops in the center of the HAfT19 RNA structure (large brackets) and a subtle rearrangement of the terminal structure (small brackets). Cloning and complete sequencing of HAfT genes and splice variants will eventually reveal more accurate predictions of HAfT transcript structures.

### HAfT variants

**Fig 5:**
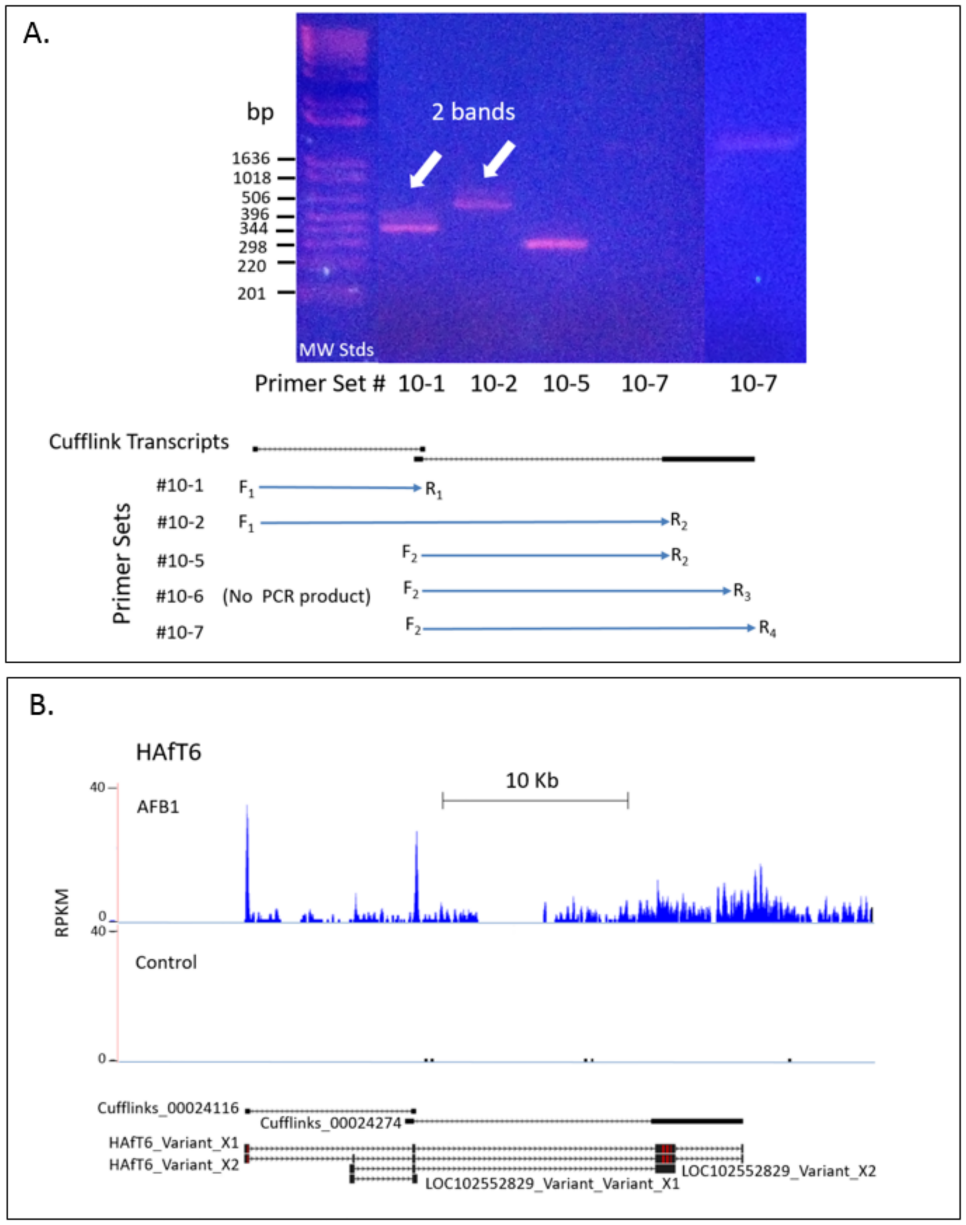
PCR cloning clarifies HAfT6 transcript sequence and structure. The structure of HAfT 6 was studied by PCR cloning and Sanger sequencing. The overlap of Cufflinks transcripts (Cufflinks_00024116 and 00024274) suggested a longer, more complex transcript. Primer sets were designed using sequences from both Cufflinks transcripts to test if they comprised a longer single transcript. In Panel A, several primer sets spanned different portions of the two Cufflinks transcripts at this locus. Individual PCR products shown in the agarose gel were excised separately, cloned and Sanger sequenced. Primer Set#7 was amplified twice to clearly show a PCR product (far right lane). In Panel B, the combined consensus Sanger sequences from all primer sets showed two variants, X1 and X2, containing either four or five exons, respectively. Note that red bands in black exons indicate Sanger sequence base variants that differ from alignment with Rn6. See text for further details.

Multiple variants were observed for eight HAfT transcripts including HAfT6, 7, 8, 10, 19, 21, 22 and 23 based on experimental Sanger sequencing data. Three to five *E*. *coli* clones were isolated for forward and reverse sequencing from PCR and RACE products and at least 3 concordant sequences were observed for each variant. HAfT6 (Fig 5) is representative of the challenges in Cufflinks assembly for some HAfT transcripts. Spikes in RNA-Seq reads were not observed for each exon in the HAfT6 locus, so we hypothesized that the two overlapping assembled transcripts, Cufflinks_00024116_528nt and Cufflinks_00024274_5559nt, were actually one transcript. Fig 5, Panel A shows the PCR primer strategy based on sequences of the two Cufflinks transcripts. Primers were designed to test the hypothesis that the two Cufflinks transcripts could be bridged into one transcript. Some of the primer sets (#10–1, #10–2) produced two amplicons, indicative of splice variants. Each band for Primer Sets#1 and #2 was excised and sequenced separately, revealing two variants X1 and X2 that differed by one additional exon. For Primer Set#5, only one PCR band was clearly observed. Primer Set#7 was amplified twice to clearly show a PCR product (1μL versus 2μL RT reaction product in the PCR reaction). Upon sequencing the PCR product from Primer Set#7, results showed that the long second exon of Cufflinks_00024274 was in fact two exons. Consistent with this finding, Primer Set#6 did not produce a PCR product suggesting that no exon occurred at the R3 primer site. It worth noting that only one PCR product was observed for Primer Sets#5 and #7, since their amplified sequences were downstream of the unique variant exon observed with Primer Sets#1 and #2. Fig 5, Panel B shows the compilation of Sanger sequencing data to form HAfT6_VariantX1 and VariantX2. HAfT6 variants shared sequences with variants predicted for the lncRNA, LOC102552829 with greater similarity to LOC102552829_VariantX2. Our sequencing data also adds new information to the predicted lncRNA sequences (LOC102552829_VariantX1 and X2) by demonstrating the presence of novel first and last exons not found in LOC102552829. HAfT6_VariantX1 contains 4 exons while HAfT6_VariantX2 has 5 exons. Further study of HAfT6 will likely reveal additional splice variants in liver and other tissues and insights into differential function.

Other HAfTs suggest that adjacent lncRNAs can sometimes be part of a larger transcript as shown by HAfT8 (S1 Fig). HAfT8 Variant_X1 and X2 span sequences of the predicted lncRNAs, LOC102554927 and LOC103694863, sharing two exons of each lncRNA while skipping other exons. HAfT8 Variant_X3 includes an exon of LOC102554927 but also contains a new exon. The estimated length for the genomic loci for HAfT8 variants X1 and X2 is about 30kB but the exact sequence and number of variants will require further work.

### HAfT orientation and proximity to adjacent genes

**Fig 6:**
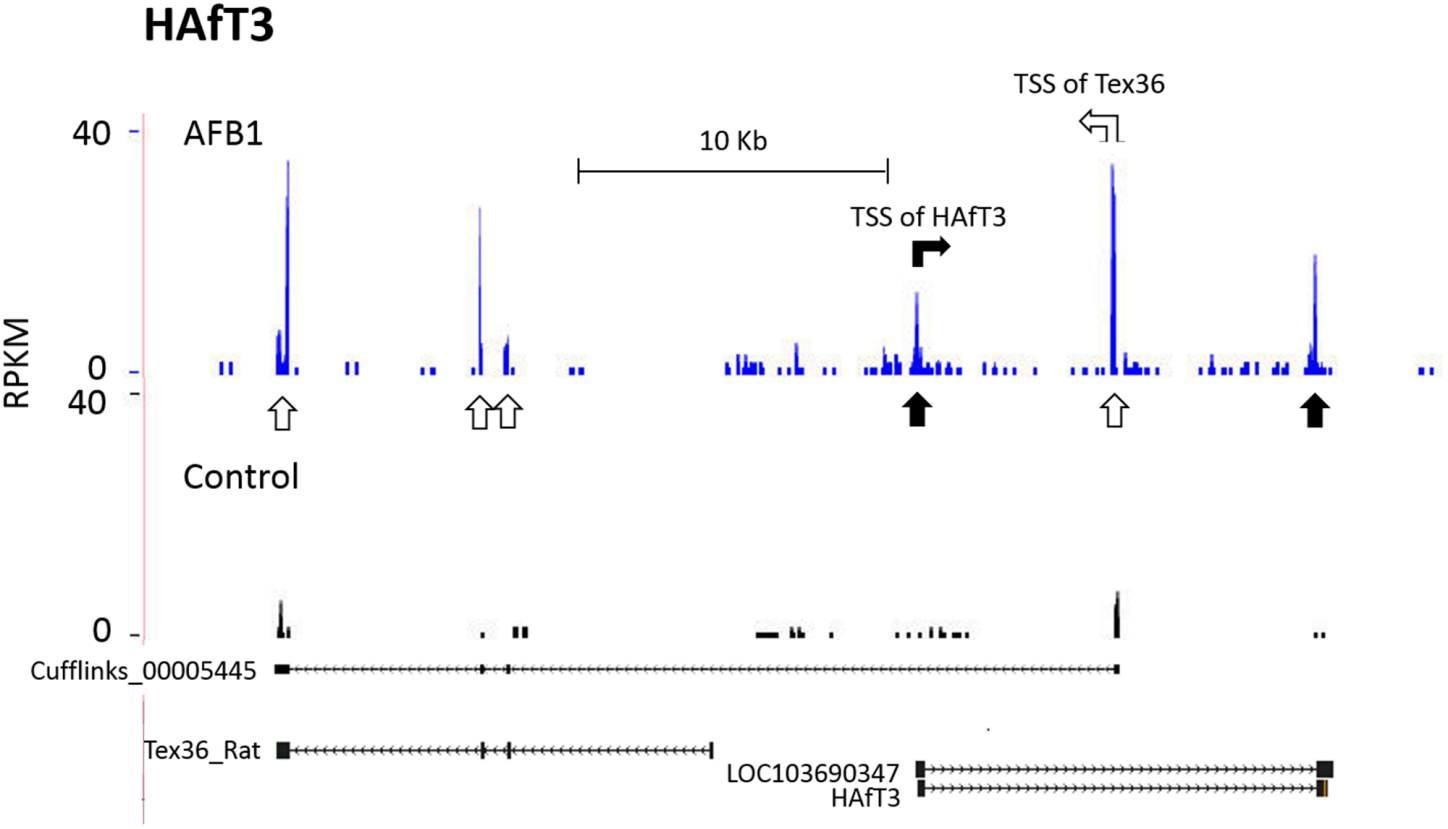
Antisense lncRNA HAfT3 overlaps Tex36. Primers to transcript Cufflinks_00005778 on Chr1 show only 2 exons (solid arrows) were transcribed on the opposite strand in an antisense direction to the rat Tex36 homolog (also known as LOC499279) as shown by open arrows for each of 4 exons. A new predicted start site (TSS) for Tex36 that overlapped HAfT3 was evident from RNA-Seq reads that aligned with Cufflinks_00005445 and was missing from the predicted Tex36 transcript (NM001024288) for rat.

The orientation of HAfT transcripts to other genes may be of functional importance (S4 Table). For example, HAfT3 (Fig 6) is on the forward (+) strand and in antisense orientation to the protein coding transcript, Tex36 (LOC499279). Display of RNA-Seq reads does not account for allelic orientation, so the genome browser track for HAfT3 gives the appearance of a 3-exon transcript. However, PCR-based cloning and sequencing showed only two exons could be found for the HAfT3_764nt transcript. The sharp spike of reads between exons 1 and 2 of HAfT3 is apparently a new starting exon 1 of Tex36 that maps in the forward direction. Evidence of opposite orientation for HAfT3 and Tex36 comes from the assembly of RNA-Seq reads. The assembly of Cufflinks_00005445 for Tex36 shows a 4-exon transcript on the reverse (-) strand where the primary difference from the predicted LOC499279 for Tex36 is a new transcriptional start site (TSS) for exon 1. Notably, AFB1 exposure significantly increased expression of both HAfT3 and Tex36 transcripts by 4.6-fold and 4.4-fold, respectively.

In addition to HAfT3, other HAfTs were examined for their orientation (+, or -) to immediately adjacent, annotated genes and for any expression changes (S4 Table). There were several expression-altered, HAfT-adjacent genes that included: Ephb3 and HAfT4 (sense orientation); Bicc1 and HAfT13 (antisense orientation); Inhba and HAfT19 (antisense orientation); Cdkn1a and HAfT21 (sense orientation); A1bg and HAfT22 (antisense orientation); mdm2 and HAfT23 (sense orientation); and A1bg and HAfT25 (antisense orientation). Of the expression-altered, annotated genes that were adjacent to HAfTs, there were five antisense HAfT-gene pairs compared to three sense HAfT-gene pairs.

HAfTs in the 7q33 chromosomal region are of high interest due to the presence of Myc, a cell proliferation gene. Sufficient data was obtained from PCR and RACE products to obtain a consensus sequence for HAfT25 that was most homologous to the hypothetical rat Pvt1 VariantX2 (See S3 Fig). Pvt1, a known regulator of Myc [40], is adjacent to Myc at 60Kb. HAfT22 (7.2-fold increase) is in the same 7q33 region as HAfT25 (Pvt1) at 0.5Mb distance from Albg (10.5-fold increase) and 0.8Mb from Myc (no expression change). Also on chromosome 7 at q22, we found HAfT23 was highly upregulated at 660-fold increase and was nearby mdm2 (0.3Mb) that increased 2.8-fold while mdm1 expression at 0.1Mb distance away was unchanged. Adjacent genes to HAfTs and their expression changes (observed in prior RNA-Seq work [26]) are summarized in S4 Table.

### Mouse and human homology

**Fig 7:**
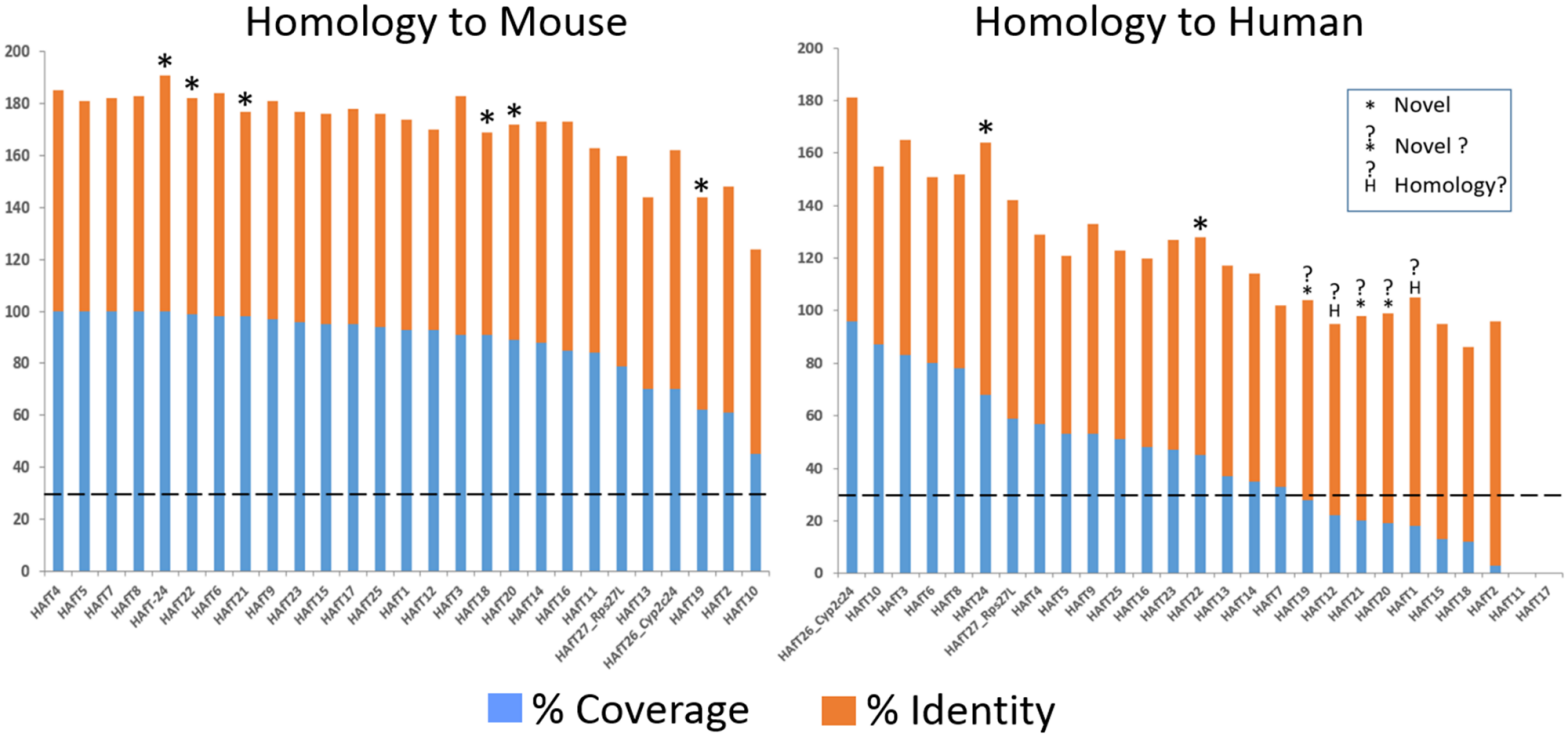
HAfT homologies to mouse and human genomes. All transcripts from Table 1 were searched for homologous mouse or human chromosomal regions. Each HAfT locus was grouped and then sorted by the highest percent coverage at a chromosomal locus for mouse (MoHomolChrRegions tab) and for human (HuHomolChrRegions tab) as shown S5 Table. Results for a representative transcript at each HAfT locus are graphed in Fig 7. A 30% sequence coverage (dotted line) was set as the criterion for rat transcript homology to a mouse or human chromosomal locus. All rat HAfTs aligned with mouse homologous loci including 6 novel lncRNAs (*) without RefSeq annotation in rat or mouse. For human chromosomal loci, there were 17 rat HAfTs aligning above the 30% coverage criterion from which 2 were known genes (Cyp2c24, Rps27l) and 2 had potentially novel human homologs (*). Another 5 human homologs that were below the 30% coverage cutoff that included 3 potentially novel (?*) human homologous loci (HAfT 19, 20 and 21) and 2 loci (e.g. HAfT1 and HAfT12) that aligned to annotated rat lncRNAs (?H) based on genomic synteny (see Table 2).

Alignment searches were conducted in the chromosomes of mouse and human genomes for all HAfT transcripts and results (p<0.01) are described in S5 Table. Fig 7 shows the percentage coverage and identity values for a representative transcript sorted by the highest percent coverage by BLASTn at each HAfT locus in mouse (see MoHomolChrRegions tab, S5 Table) and human (see HuHomolChrRegions tab, S5 Table). The data show that all rat HAfTs had homologous loci in mice, including novel, unannotated HAfTs (asterisk). As expected, relatively high homologies were found for rat and mouse as well as rat and human coding genes for Rps27L and Cyp2c24. Similarly, Fig 7 shows that all rat HAfTs were homologous with mouse chromosomal loci at ≥50% sequence coverage (except HAfT10 at 45%).

Fig 7 also shows fifteen human homologous genomic loci to rat HAfTs that met a 30% coverage criterion (dotted line). Included in these fifteen human loci, were homologous loci to two novel unannotated rat transcripts − HAfT22 (45% coverage; 83% identity) and HAfT24 (68% coverage; 96% identity). A search of the NCBI EST database showed evidence of transcription for all novel mouse and human transcripts except for absence of mouse ESTs for HAfT21 or human ESTs for HAfT1 and HAfT20.

Evidence for possible homologous transcripts is genomic synteny. HAfTs in Fig 7 were first aligned to a mouse and human genomic locus; then that genomic locus was compared for similarity of surrounding transcripts. (Table 2). Rat HAfTs mapped with a high degree of similarity for surrounding transcripts in the mouse genome, usually within 1 to 2 Mb, consistent with their >50% sequence homology. Similarly, when rat HAfTs were mapped to the human genome, those HAfTs with >30% sequence alignment occurred at a syntenic human genomic locus. Of those remaining HAfTs that had less than 30% sequence alignment, there were five HAfts (HAfT1, 12, 19, 20 and 21) that had substantial amounts of sequence aligned in the human genome at syntenic loci to rat and mouse genomes. Thus, HAfT19, 20 and 21 appear to align to novel human transcriptional loci (indicated by ‘?*’ in Fig 7) and HAfT1 and HAfT12, while not novel rat transcripts, align to potentially homologous human transcriptional loci (indicated by ‘?^H^’ in Fig 7) for lncRNAs. All transcripts in syntenic loci in Table 2 are shared by rat, mouse and human genomes when a chromosome number is indicated. We observed that only five HAfTs (HAfT2, 11, 15, 17 and 18) showed little human sequence homology (<30% homology) and no syntenic loci (indicated by ‘Human–NO Synteny’ in Table 2).

**Table 2:** Synteny of HAfTs in mouse and human chromosomes^a^.

HAfT#	Species, Distance, Chromosome#	Surrounding Genes and Transcripts at Each Locus, Conserved in Both Mouse and Human (Genes Upstream, Top row; Genes Downstream, Bottom row)									
**HAfT1**	Mouse 1 Mb; Chr19	Tcf7l2	Vti1a	Zdhhc6	Acsl5	Gucy2g					
	Human 1 Mb; Chr10	Habp2	Nrap	Casp7	Plekhs1	Dclre1a	Nhlrc2	Adrb1			
**HAfT2**	Mouse 1Mb; Chr14	Tpt1	Slc25a30	Cog3	Spert	Siah3	Zc3h13	Cpb2	Lcp1		
	Human—NO Synteny	Gtf2f2	Kctd4	Gpalpp1	Nufip1	Tsc22d1					
**HAfT3**	Mouse 1 Mb; Chr7	Tex36	Ctbp2	Zranb1	Zc3h13	Ctpb2					
	Human 1 Mb; Chr10	Edrf1	Uros	Mmp21	Bccip	Dhx32	Fank1	Adam12			
**HAfT4**	Mouse 1Mb; Chr16	Ephb3	Chrd	Ece2	Dvl3	Camk2n2	Eif2b5	Alg3	Fsmd2	Abcf3	Ap2m1
	Human 1Mb; Chr3	Ehhadh	Map3k13	Thpo	Polr2h	Clcn2					
**HAfT5**	Mouse 2Mb; Chr8	Thap1	Hook3	Fnta	Pomk	Ash2l					
	Human 4Mb; Chr8	Zfp703	Erlin2	Prosc	Brf2	Adrb3	Got1l1	Rab11fip1	Eif4ebp1	Chrnb3	Chrna6
**HAfT6**	Mouse 2Mb; Chr8	Fgfr1	Letm2	Ash2l	Star	Lsm1	Bag4	Dhd2	Plpp5		
	Human 3Mb; Ch8	Tacc1	Htra4	Tm2d2	Adam9	Adam32	Adam5	Adam3a	Ido1		
**HAfT7**	Mouse 1Mb; Chr13	Diras2	Syk	Auh	Nfil3						
	Human 1Mb; Chr9	Gadd45g	Sema4d	Secisbp2	Cks2	Shc3	S1pr3				
**HAfT8**	Mouse 3 Mb; Chr13	Cd83	Rnf182	Mcur1	Sirt5	Gfod1	Tbc1d7	Phactr1	Adtrp		
	Human -3Mb; Chr6	Tfap2a									
**HAfT9**	Mouse 4Mb; Chr18	Zfp608	Csnk1g3	Cep120	Ppic	Snx24	Snx2	Sncaip	Znf474	Lox	
	Human 4Mb; Chr5	Gramd3	Aldh7a1	Phax	Tex43	Lmnb1	March3				
**HAfT10**	Mouse 2Mb; Chr18	Map3k1	Mier3	Gpbp1	Actbl2						
	Human 2Mb- Chr5	Ankrd55	Il16st	IL13lra	Ddx4	Slc38a9	Plpp1	Skiv21			
**HAfT11**	Mouse 1Mb; Chr8	Urb2	Taf5	Abcb10	Acta1	Ccsap	Rab4a	Gas8			
	Human—NO Synteny	Galnt2	Pgbd5	Cog2	Agt	Capn9	Ttc13	Arv			
**HAfT12**	Mouse 1 Mb; Chr3	Mttp	Trmt10a	Adh7	Adh1	Adh6	Adh4	Adh5	Metap1	Eif4e	
	Human 1 Mb; Chr4	Dapp1	Lamtor3	Dnajb14	H2afz	Ddit4l2	Emcn				
**HAfT13**	Mouse 4Mb; Chr10	Tfam	Ube2d1	Cisd1	Ipmk	Zwint					
	Human 4Mb; Chr10	Bicc1	Phyhip1	Mrln	Ank3						
**HAfT14**	Mouse 2Mb; Chr4	Nfib	Mpdz								
	Human 2Mb; Chr9	Zdhhc21	Cer1	Frem1	Ttc39b	Snapc3	Psip1	Bnc2			
**HAfT15**	Mouse 3Mb; Chr12	Tmem179	Aspg	Rd31	Klc1	Bag5	Apopt1	Zfyve21	Ppp1r13b	Mta1	
	Human—NO Synteny	Siva1	Akt1	Zbtb42	Pld4	Cdca4	Gpr132	Nudt14	Brf1	Btbd6	
**HAfT16**	Mouse 3Mb; Chr9	Fat3	Mtnr1b	Slc36a4	Vstm5	Taf1d	Cep295	Med17	Fanx1	Hephl1	
	Human 3Mb; Chr11	Chordc1	Naa1ad2								
**HAfT17**	Mouse 1Mb; Chr7	Tsku	B3gnt6	Capn5	Myo7a	Pak1	Aqp11	Clns1a			
	Human—NO Synteny	Gucy2d	Lrrc32	Prkrir	Wnt11	Uvrag	Dgat2				
**HAfT18**	Mouse 1Mb; Chr2	Cdnf	Hspa14	Suv39h2	Dclrelc	Meig1	Olah	Acbd7	Rpp38	Nmt2	
	Human—NO Synteny	Fam107b	Frmd4a								
**HAfT19**	Mouse 2Mb; Chr13	Inhba	Gli3								
	Human 2 Mb; Chr7	Sugct	Cdk13	Mplkip	Rala	Yae1d1					
**HAfT20**	Mouse 1Mb; Chr18	Commd10	Cdo1	Atg12	Ap3s1	Tmed7	Fem1c	Ticam2			
	Human 1Mb; Chr5	Sema6a									
**HAfT21**	Mouse 1Mb; Chr17	Srsf3	Stk38	Pnp1a1	Brpf3	Mapk13	Mapk14	Srpk1	Lhfpl5	Clpsl2	Clps
	Human 1Mb; Chr6	Cdkn1a	Cpne5	Ppil1	Pil6	Mtch1	Fgd2				
**HAfT22**	Mouse 4Mb; Chr15	Myc	Gsdmc	Fam49b	Asap1						
	Human 4Mb; Chr8	Trib1	Sqle	Trmt12	Rnf139	Ndufb9	Mtss1				
**HAfT23**	Mouse 2Mb; Chr10	Mdm1	IL22	Ifng	Dyrk2						
	Human 2Mb; Chr12	Rap1b	Nup107	Slc35e3	Mdm2	Cpm	Cpsf6				
**HAfT24**	Mouse 2Mb; Chr11	Lhx1	Aatf	Acaca	Dusp14	Tada2a					
	Human 2Mb; Chr17	Mrm1	Dhrs11	Ggnbp2	Figw	Myo19					
**HAfT25**	Mouse 1 Mb; Chr15	Myc	A1bg								
	Human 1 Mb; Chr8	Pvt1									

^a^A representative transcript for each HAfT was selected for synteny determination in the mouse (NCBI37/mm9) or human (GRCh37/hg19) genomes. A 1-2Mb genomic distance was usually examined but occasionally 3-4Mb lengths were examined for surrounding upstream and downstream genes on either side of each HAfT, shared in mouse and human. Relevant syntenic chromosome number is indicated for mouse or human in column 2.

### Functional motifs and sequence homologies

LncRNAs are non-coding by definition but many contain sizeable ORFs that can sometimes be translated [41], so we queried HAfT sequences in the conserved domain database (CDD) and results are summarized in S6 Table. Domains were easily detected for coding genes Cyp2c24 and Rps27L, that contain cytochrome P450 family and ribosomal protein family domains, respectively. There are two HAfTs with sizeable open reading frames (ORFs), HAfT1 and HAF5, that could produce hypothetical proteins that do not have detectable conserved domains. The ORFs for HAfT1 and LOC103369380 could produce polypeptides of 122aa and 115aa, respectively (aa, amino acids). A 226aa polypeptide has been predicted for the HAfT5 ORF, classified as a hypothetical coding transcript. In addition, a search of other HAfTs shows specific transcripts that contain multiple domains of interest within defined coding sequence regions. For example, HAfT8_VariantX3 shows homologies to ATP synthetase and GNS1/SUR4 (long chain fatty acid elongation) as well as two families of unknown function (DUF3796; DUF1754) (See S6 Table). HAfT17 has homologies to Replication Protein A family and HAfT18 with homologies to the RRN3 superfamily (RNA polymerase I transcription initiation factor) and the transcriptional regulator, CsoR-like_DUF156 superfamily.

HAfT functions may also be indicated by other defined sequences that are homologous with regulatory portions of annotated genes. For example, the first exon (147nt) of HAfT19 variants, X1, X2 and X3, has an unusually high homology at 92% with a sequence in 3’ UTR region of the gene, Prrc1. Search of this HAfT exon1 sequence shows alignment to rno-miR-105, inferring possible miR regulation. In addition, repetitive sequences may have functional and secondary structure consequences for lncRNAs.

RepeatMasker algorithm [38, 42] was used to find interspersed and low complexity repeats of all HAfT related sequences and search results are shown in S7 Table. Notably, short tandem repeats (STRs) of 2 to 6 nucleotides in length were found in five experimental sequences from PCR and RACE products (HAfT1, 5, 6, 8 and 23), in twelve of the predicted lncRNA loci and in thirteen of the Cufflinks assembled transcripts. In the case of HAfT6 in Fig 5, both X1 and X2 variants of HAfT6 share the last two exons (Sanger sequence verified) that contain two different repetitive elements, (CA)n and (GTGGTT)n of 32nt and 42nt length, respectively. A long GATA repeat was found in HAfT16 in the Cufflinks_00055698 transcript and predicted locus LOC102553833.

## Discussion

AFB1 treatment in rats produced differential expression of several, unannotated hepatic transcripts from RNA-Seq data described as HAfTs in our original report [26]. A reanalysis of these sequences in NCBI databases showed a total of 25 unknown transcripts, since sequence alignment identified two protein-coding genes (Cyp2c24, Rsp27L). The current study attempted to validate and identify these novel transcripts from liver RNA extracted from AFB1 exposed animals. To our knowledge, HAfTs are distinct from chemically-responsive rat lncRNAs found by others (e.g. [33]). We used Sanger sequencing of cDNA after PCR cloning and by 5’- and 3’-RACE with future studies planned for full length sequencing and quantitative analysis studies of HAfT genes in various tissues.

Detailed database searches of consensus transcripts from Sanger data and Cufflinks transcripts revealed that most HAfTs are lncRNAs. HAfT1 to 17 (except HAfT5) were predicted as lncRNAs by Gnomon, described by NCBI as an automated gene prediction tool [43]. Updated transcriptome annotations of various eukaryotic species are periodically produced by NCBI upon the application of Gnomon to deep sequencing datasets. For the rat transcriptome, *Rattus norvegicus* annotation release 106 was supported by Gnomon analysis of pooled RNA sequencing datasets from female BN/SsNHsdMCW rats and one SHR strain male (BioProject PRJNA12455) as representatively described in LOC nucleotide records (e.g. LOC103691380 and others in Table 1). Gnomon transcript models either have no predicted CDS (coding sequence) or only a short CDS with no supporting alignments and are generally annotated as non-coding transcripts. HAfT5, however, is registered as LOC103693988, a protein coding transcript for a hypothetical product of 226aa, predicted by the Rat Genome Database (https://rgd.mcw.edu). HAfT1 LOC103691380 sequence has ORFs that could be protein coding with potential polypeptides of 122aa and 115aa in length, respectively, but further work will be needed to verify possible translation products. The remaining transcripts, HAfT17-25, also qualify as lncRNAs since they are >200nt, do not have obvious ORFs of size, and generally are without recognizable functional domains [44].

Here, we now report six HAfTs—HAfT18, 19, 20, 21, 22 and 24—as novel rat lncRNAs that are not found in the rat annotation release 106. Several reasons may account for these findings. RNA-Seq data from different rat strains were used by Gnomon compared to our RNA-Seq study [26] in which F344 strain rats were examined. Further, a predominance of female rats was used to annotate release 106 whereas our study only used male rats that may involve hormonal differences in expression. Perhaps more importantly, some HAfT transcripts were induced by AFB1 far above controls at levels not ordinarily observed in unchallenged animals. For example, AFB1 treatment induced HAfT20 and HAfT24 transcripts to measurable reads starting from either low or no reads in control animals. It could be argued that increased sequencing for greater depth of coverage might have detected otherwise low copy transcripts, but RNA-Seq of cell and organ-specific transcriptomes suggest there are silent portions of the transcriptome that await activation upon appropriate stimuli [45, 46] or chemical exposure [47]. In addition, our Sanger sequencing data suggests that short read transcriptomic data may create challenges for precise mapping and transcript prediction algorithms even when sufficient sequence reads may be present in all samples (e.g. HAfT18, 19, 21 and 22). Our strategy of using primer-based PCR to improve computational transcript models is similar to other researchers that have reported refinements in the human transcriptome [48], for various disease state transcriptomes like pulmonary fibrosis [49] and in the transcriptomes of other species including equine [50], avian [51] and murine [52] species.

Detection of splice variants are often observed but functional consequences may not be immediately known. In some cases (e.g. KRAS and TP53), such transcript variants can be important in cell signaling and development of disease [53, 54]. In this study, Sanger sequencing data provided evidence for several splice variants including eight different HAfTs (Table 1). We anticipate the finding of additional splice variants for the HAfTs described here when other extrahepatic tissues are eventually explored as has been documented for other lncRNAs [55, 56]. Like coding genes, multiple lncRNA variants provide opportunities to assume differing shapes and structures, binding properties, and biological functions [57, 58]. The lncRNA HOTAIRM1 is transcribed into unique sequence variants with preferential binding properties to either UTX/MLL or PRC2 complexes in regulating expression of the developmentally important HOXA genes [58]. The cancer susceptibility candidate 18, CASC18, is a lncRNA in a cancer predisposition locus at 12q23.3 and was recently cloned as 4 splice variants (A, B, C, and D)[58]. Only CASC18-D expression increased during neural differentiation that was coincident with enhanced PAX6 expression as an established neural marker, suggesting a crucial role of variant D in neural differentiation [58]. In our study, three experimental variants of the novel HAfT19 were found, of which two transcripts, VariantX2 and X3, may occur from exon skipping that could have functional consequences. Therefore, establishing baseline expression for HAfT transcripts and variants compared to the HAfT profiles during AFB1 treatment could lead new preneoplastic markers before the onset of hepatocellular carcinoma.

Gene homologies among species are generally driven by DNA and amino acid sequence homologies for protein-coding genes while lncRNA sequences are generally less conserved by comparison. lncRNAs are believed to evolve more rapidly than coding-genes, may not always have direct orthologs in other species, and may be selected for conserved secondary and tertiary structures [59, 60]. One proposal is to evaluate lncRNA conservation as a matter of four dimensions that include sequence, structure, function and expression from syntenic loci, looking to Hotair and Malat1 lncRNAs as examples [61]. We took a similar approach by examining HAfT sequences and expression from syntenic loci in looking for potential homologs. All 25 rat HAfTs had corresponding homologous loci (e.g. homologs) in mice that included 23 lncRNAs and 2 possible coding RNAs, based on >50% sequence homology and genomic synteny. For the human genome, we propose the 15 human homologous loci HAfTs represent lncRNA homologs based on sufficient sequence homology and synteny; and another possible 5 HAfTs (see Fig 7) may also have corresponding human transcripts with weaker homologies but retaining synteny among the 3 species. Cyp2c24, Rps27l have related human homologs. Thus, only five rat HAfTs (HAfT2, 11, 15, 17 and 18) showed little evidence for having homologous loci in humans.

Short tandem repeat (STR) sequences were found in HAfTs and predicted lncRNAs. This observation suggests that other sequence features besides conventional functional domains are likely important for lncRNA structure and activity. STRs are patterns of two or more nucleotides repeated in tandem of varying lengths and complexity, and underlie several neurodegenerative diseases such as Huntington’s disease and Fragile X syndrome [62, 63]. The occurrence of STRs within genes can affect transcript secondary structure and RNA function [64, 65]. For example, the Firre (functional intergenic repeating RNA element) lncRNA has several STRs such as the RRD repeat that functions as a ribonucleic nuclear retention signal and the R0 repeat that acts as a DNA enhancer element [66]. The lncRNA CAT7 has a unique tandem repeat domain that plays a critical role in its interference with PRC1 (polycomb in repressive complex 1) during embryogenesis [67]. The STR TAAAn repeat in the promoter region of the lncRNA PCA3 (prostate cancer gene antigen) has been found as a risk factor for prostate cancer [68]. Gomafu is a lncRNA with a distinctive tandem repeat that allows binding to the SF1 splicing factor to affect splicing efficiency within the nucleus [69]. Finally, transcripts with GATA repeats (as observed in HAfT16) have enhancer repressor activity in Drosophila and human cells to finely regulate gene transcription [70].

Finding evolutionary relationships and conserved regions in lncRNA sequences represent acknowledged challenges. For many lncRNAs, their functions are often linked to their secondary and tertiary structure so that considerable sequence variation and splice variants may occur among species to achieve the structural requirements needed for activity [71]. Pvt1 is a lncRNA with pleiotropic functions that include miRNA sponging activity; inhibition of Myc phosphorylation and degradation to prolong Myc activity; enhancement of the Tgfβ pathway; formation of novel Pvt1 fusion transcripts; and transcription of miR-1200 family members [40, 72, 73]. Pvt1 transcripts have not been characterized in rat liver, although there are 21 variants predicted for the rat Pvt1 gene by Gnomon. Here, we found primarily one liver transcript, HAfT25, and it was most closely aligned with the predicted Pvt1_VariantX2 (XR593559). HAfT25 has a relatively high homology with mouse Pvt1 (94% coverage, 82% identity) but less so when compared to human Pvt1, at 51% coverage and 72% identity. Pvt1 is upregulated in a variety of tumors and supports increased cell proliferation and inhibition of apoptosis, most frequently by upregulating the Myc oncogene [40]. In our study, HAfT25 was overexpressed by 51-fold (Fig 1) after 90-day treatment with AFB1 prior to development of hepatocellular carcinomas, but Myc transcript expression was not increased, suggesting HAfT25 may engage other pathways involved in transformation in the preneoplastic rat liver.

Coregulation of lncRNAs and neighboring genes is an area of active research in understanding lncRNA functions. For example, 138 coexpressed lncRNA–mRNA pairs were identified from differentially expressed lncRNAs and mRNAs in human glioblastomas [74] and 63 co-regulated lncRNAs and mRNAs were found in human breast cancer spheroids [75]. In our study, the overlapping transcripts, HAfT3 and Tex36 (unknown function), and their concurrent increased transcription suggest they may be a linked gene pair for coexpression. Gene coexpression is observed during development of many organisms [76] and occurs with greater frequency in some tumors [74]. All together there are 8 possible gene pairs by which HAfTs may influence expression of neighboring genes (S4 Table). Interesting gene pairs for further study that might be relevant to transformation are, HAfT13 and the developmental gene, Bicc1 [77]; HAfT21 and the cell cycle inhibitor, cdkn1a [78]; and HAfT23 and the E3 ubiquitin ligase/protooncogene, mdm2 [79]. Elevated expression of mdm2 is of particular interest since AFB1 also increases Rps27l expression. RPs27l plays a critical role in regulating cell proliferation and apoptosis via the mdm2-p53 axis [80] and also by enhancing expression of DNA repair enzymes [81]. Additionally, another lncRNA–PRAL (p53 regulation-associated lncRNA)–plays a protective role against genomic damage by stabilizing p53 from Mdm2-mediated ubiquitination through its three stem-loop motifs [82]. These transcriptional changes are consistent with activation of several integrated cell defense modules involving lncRNAs in response to continual genotoxic stress by AFB1.

## Conclusions

In this study we provide Sanger sequencing data that HAfT transcripts are primarily lncRNAs. Two possible exceptions are HAfT1 and HAfT5 that have sizeable open reading frames. We discovered 6 novel rat HAfTs without prior annotation that also meet criteria as lncRNAs. All rat HAfTs, including novel HAfTs, have a corresponding mouse homolog or a homologous locus in mm9 based on ≥50% alignment (exceeding a 30% cutoff) of rat transcripts. In addition, there were 17 HAfT transcripts that may have human homologs, with 2 potentially novel transcripts related to HAfT22 and HAfT24 and possibly others. The sizeable fold change of HAfT lncRNAs (HAfT6, 11, 20, 23, 24, 25 at >50-fold increase) could provide new preneoplastic biomarkers in rat and potentially in mouse and human liver transcriptomes. Since a rat homolog for Pvt1 (lncRNA oncogene) was among these highly induced HAfTs, perhaps other HAfT transcripts may be indicators of preneoplastic disease that have roles in malignant transformation, being driven by continual genotoxic stress of AFB1 exposure. Functions of individual HAfTs await further investigation after their complete sequencing and cloning by using overexpression-knockdown strategies or powerful CRISPR-Cas9 systems for studying lncRNA function [83, 84]. It is likely that secondary structural changes in lncRNAs due to splicing variants and sequence motifs like short tandem repeats will be important determinants of HAfT function.

## Supporting information

S1 Fig HAfT8 is a larger transcript composed of adjacent lncRNAs. HAfT8 transcript VariantsX1 and X2 represent the combined Sanger sequences of adjacent, predicted lncRNAs, LOC102554927 and LOC103694863. HAfT8 VariantX3 includes an exon of LOC102554927 but also contains a new exon.
(TIF)

S2 Fig Rat homolog HAfT25 versus multiple Pvt1 variants. HAfT25_1501nt transcript was aligned to available rat hypothetical NCBI transcripts for Pvt1 by BLAT search function in the UCSC genome browser. RefSeq alignment for mouse Pvt1 variants (blue font) are shown above for comparison below to rat Pvt1 variants (black font). HAfT25_1501nt is most closely aligned to the NCBI variant, Pvt1_X2_3592nt. Red arrows at the bottom of the figure point to exons that are unique to HAfT25 and are different from NCBI rat Pvt1 transcripts. The first exon has a different start site than other rat Pvt1 transcripts. The last exon of HAfT25 was different than Pvt1 transcripts because of the primer limits of RACE sequencing; so the last HAfT25 exon is likely incomplete compared to the final exon of most Pvt1 transcripts. The location of HAfT25 is chr7:102,595,304–102,924,768 in the Rn6.0 genome.
(TIF)

S3 Fig Rat homolog to the Pvt1 transcript: HAfT25, control versus AFB1. HAfT25 alignment from RNA-Seq reads is displayed in the UCSC browser. Reads from each animal (AFB1 #1–4; Controls #5–8) were aligned to the rat genome. Two Cufflinks transcripts were assembled from RNA-Seq reads, but were found to be different portions of the same transcript. After combining PCR and RACE sequences, a consensus sequence of 1501nt in length was formed. Note that the first exon at the 5’-region, indicates a different starting site than the hypothetical transcript that has been predicted for rat Pvt1_VariantX2, based on homologies to human and mouse Pvt1. Other exons of HAfT25 generally agree with the predicted Pvt1_VariantX2 rat transcript model. Note that a transcript (LOC257642) for rRNA promoter binding protein (box, arrow) appears in the Pvt1 genome browser track.
(TIF)

S1 Table Genomic locations of HAfTs. (XLSX)

S2 Table Primer sets for HAfTs. (XLSX)

S3 Table HAfT NCBI accession nos. (XLSX)

S4 Table Proximal genes to HAfTs. (XLSX)

S5 Table HAfT mouse and human homology. (XLSX)

S6 Table Conserved motifs in HAfT sequences. (XLSX)

S7 Table Repeatmasker analysis of HAfTs. (XLSX)
