# Supplementary figures and images for: HAfTs are novel lncRNA transcripts from aflatoxin exposure
Source: PLoS One. 2018 Jan 19;13(1):e0190992. doi: 10.1371/journal.pone.0190992 (PMC5774710; doi:10.1371/journal.pone.0190992)

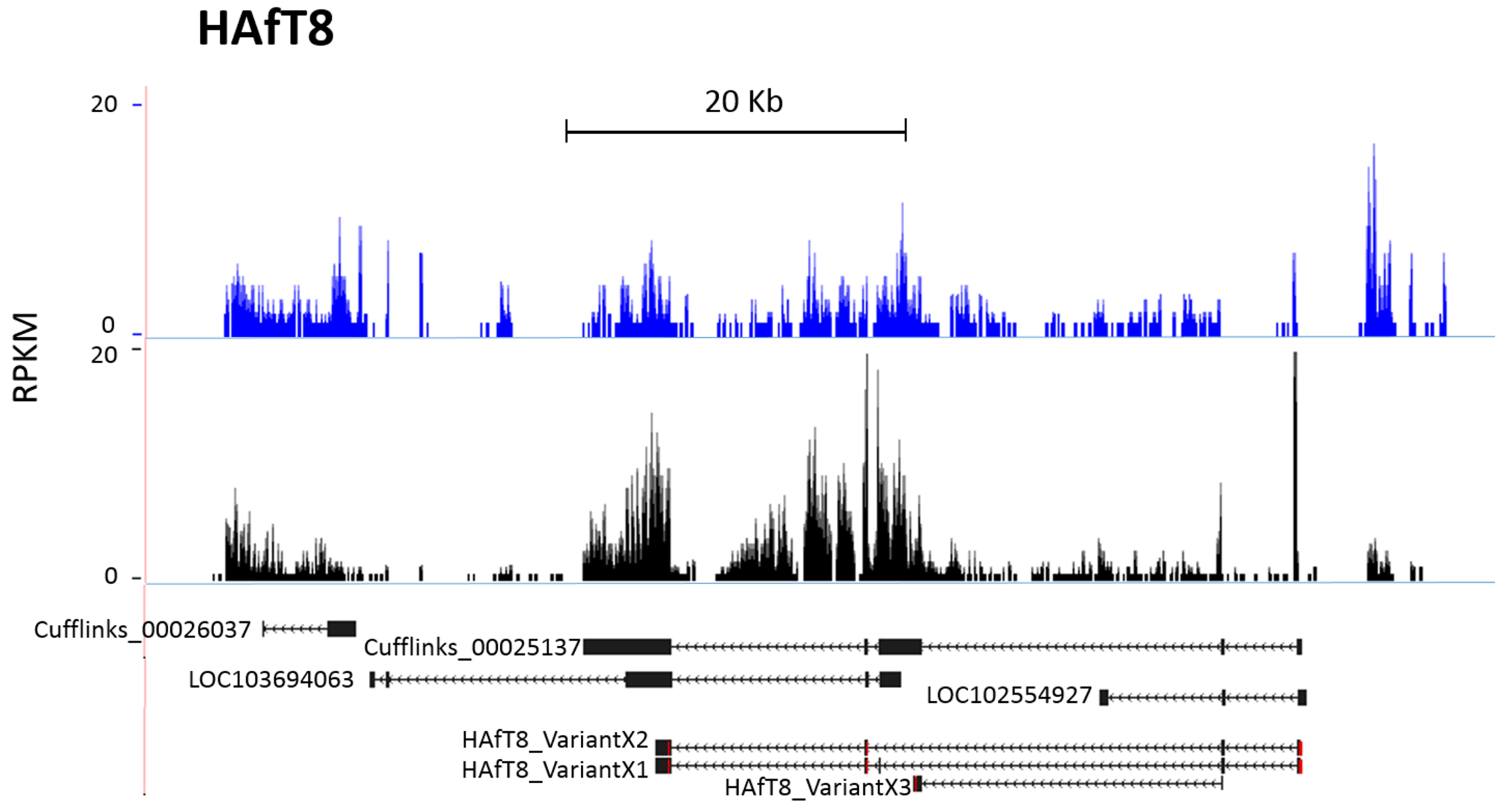

Supplement: S1 Fig — HAfT8 transcript VariantsX1 and X2 represent the combined Sanger sequences of adjacent, predicted lncRNAs, LOC102554927 and LOC103694863. HAfT8 VariantX3 includes an exon of LOC102554927 but also contains a new exon. (TIF) [file pone.0190992.s001.tif]

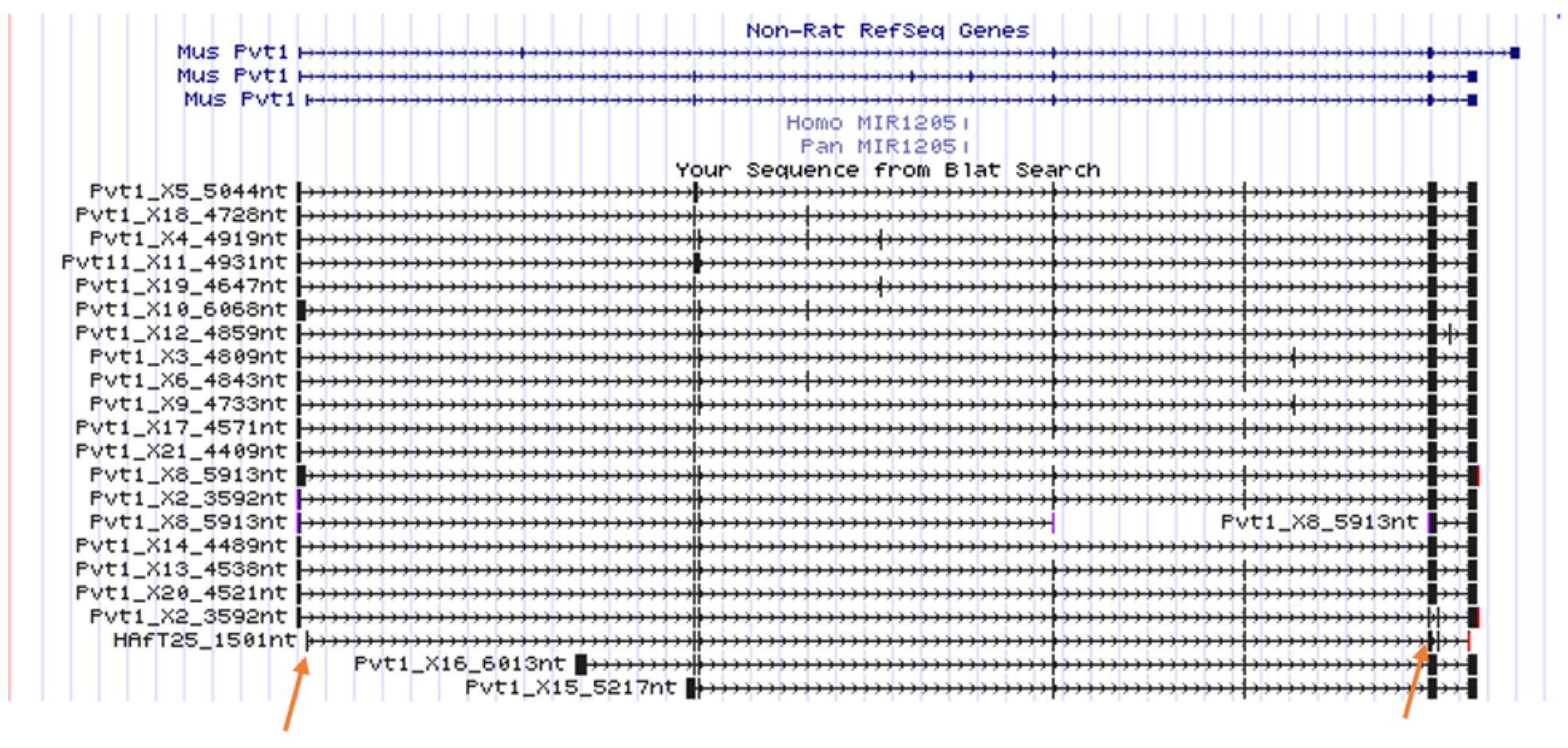

Supplement: S2 Fig — HAfT25_1501nt transcript was aligned to available rat hypothetical NCBI transcripts for Pvt1 by BLAT search function in the UCSC genome browser. RefSeq alignment for mouse Pvt1 variants (blue font) are shown above for comparison below to rat Pvt1 variants (black font). HAfT25_1501nt is most closely aligned to the NCBI variant, Pvt1_X2_3592nt. Red arrows at the bottom of the figure point to exons that are unique to HAfT25 and are different from NCBI rat Pvt1 transcripts. The first exon has a different start site than other rat Pvt1 transcripts. The last exon of HAfT25 was different than Pvt1 transcripts because of the primer limits of RACE sequencing; so the last HAfT25 exon is likely incomplete compared to the final exon of most Pvt1 transcripts. The location of HAfT25 is chr7:102,595,304–102,924,768 in the Rn6.0 genome. (TIF) [file pone.0190992.s002.tif]

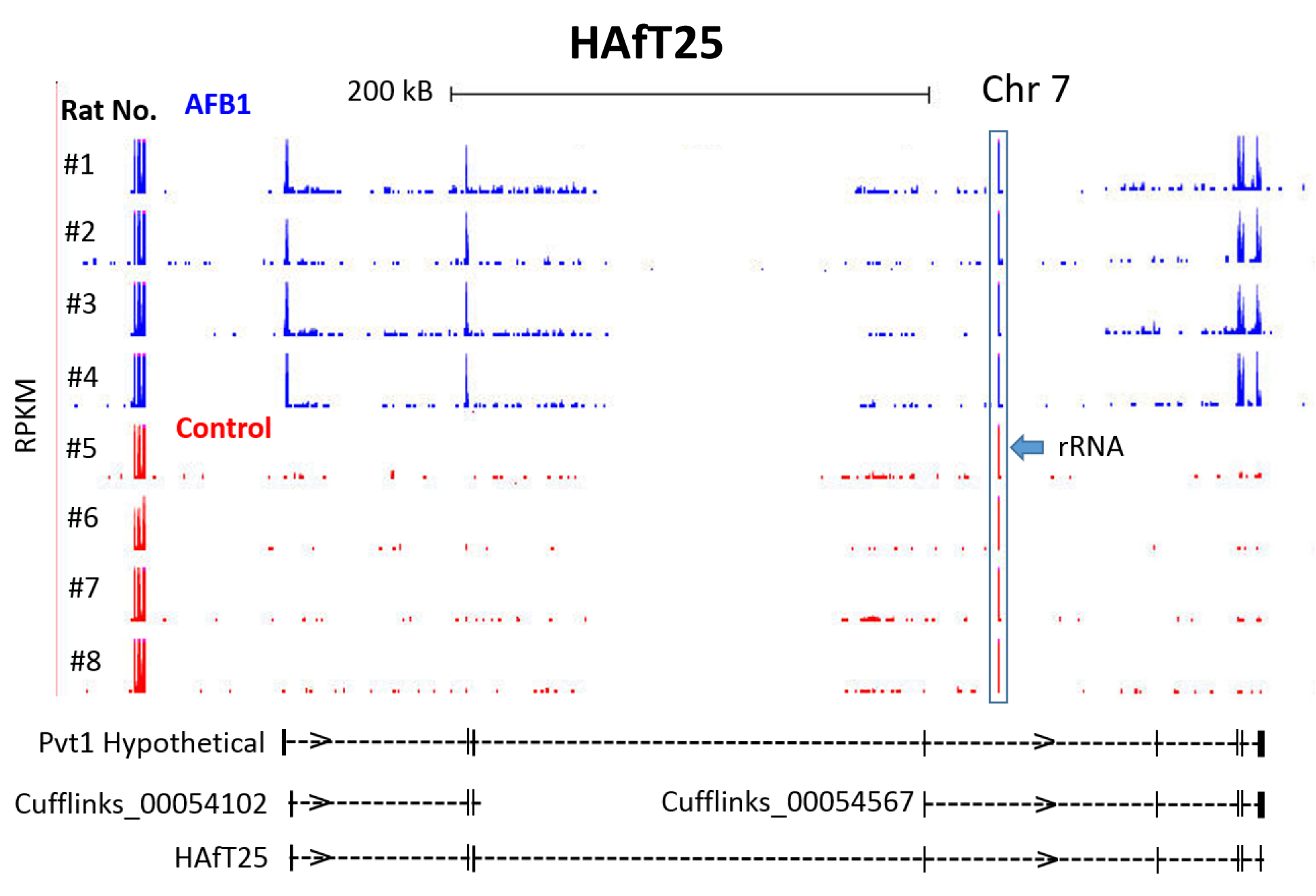

Supplement: S3 Fig — HAfT25 alignment from RNA-Seq reads is displayed in the UCSC browser. Reads from each animal (AFB1 #1–4; Controls #5–8) were aligned to the rat genome. Two Cufflinks transcripts were assembled from RNA-Seq reads, but were found to be different portions of the same transcript. After combining PCR and RACE sequences, a consensus sequence of 1501nt in length was formed. Note that the first exon at the 5’-region, indicates a different starting site than the hypothetical transcript that has been predicted for rat Pvt1_VariantX2, based on homologies to human and mouse Pvt1. Other exons of HAfT25 generally agree with the predicted Pvt1_VariantX2 rat transcript model. Note that a transcript (LOC257642) for rRNA promoter binding protein (box, arrow) appears in the Pvt1 genome browser track. (TIF) [file pone.0190992.s003.tif]
